# Quality over Quantity: How Different Dispersion Qualities of Minute Amounts of Nano-Additives Affect Material Properties in Powder Bed Fusion of Polyamide 12

**DOI:** 10.3390/ma14185322

**Published:** 2021-09-15

**Authors:** Alexander Sommereyns, Stan Gann, Jochen Schmidt, Abootorab Baqerzadeh Chehreh, Arne Lüddecke, Frank Walther, Bilal Gökce, Stephan Barcikowski, Michael Schmidt

**Affiliations:** 1 Institute of Photonic Technologies (LPT), Friedrich-Alexander-Universität Erlangen-Nürnberg, Konrad-Zuse-Str. 3/5, 91052 Erlangen, Germany; michael.schmidt@lpt.uni-erlangen.de; 2 Erlangen Graduate School in Advanced Optical Technologies (SAOT), Friedrich-Alexander-Universität Erlangen-Nürnberg, Paul-Gordan-Str. 6, 91052 Erlangen, Germany; 3 Technical Chemistry I and Center for Nanointegration Duisburg-Essen (CENIDE), University of Duisburg-Essen, Universitaetsstr. 7, 45141 Essen, Germany; stan.gann@uni-due.de (S.G.); goekce@uni-wuppertal.de (B.G.); stephan.barcikowski@uni-due.de (S.B.); 4 Institute of Particle Technology, Friedrich-Alexander-Universität Erlangen-Nürnberg, Cauerstr. 4, 91058 Erlangen, Germany; jochen.schmidt@fau.de; 5 Interdisciplinary Center for Functional Particle Systems, Friedrich-Alexander-Universität Erlangen-Nürnberg, Haberstr. 9a, 91058 Erlangen, Germany; 6 Department of Materials Test Engineering (WPT), TU Dortmund University, Baroper Str. 303, 44227 Dortmund, Germany; abootorab.chehreh@tu-dortmund.de (A.B.C.); frank.walther@tu-dortmund.de (F.W.); 7 Institute for Particle Technology, Technische Universität Braunschweig, Volkmaroder Str. 5, 38104 Braunschweig, Germany; a.lueddecke@tu-bs.de; 8 Materials Science and Additive Manufacturing, School of Mechanical Engineering and Safety Engineering, University of Wuppertal, Gaußstr. 20, 42119 Wuppertal, Germany

**Keywords:** laser powder bed fusion, polyamide 12, nanocomposites, nanoparticles, dispersion, LB-PBF, mechanical properties, additively manufactured parts

## Abstract

The great interest, within the fields of research and industry, in enhancing the range and functionality of polymer powders for laser powder bed fusion (LB-PBF-P) increases the need for material modifications. To exploit the full potential of the additivation method of feedstock powders with nanoparticles, the influence of nanoparticles on the LB-PBF process and the material behavior must be understood. In this study, the impact of the quantity and dispersion quality of carbon nanoparticles deposited on polyamide 12 particles is investigated using tensile and cubic specimens manufactured under the same process conditions. The nano-additives are added through dry coating and colloidal deposition. The specimens are analyzed by tensile testing, differential scanning calorimetry, polarized light and electron microscopy, X-ray diffraction, infrared spectroscopy, and micro-computed tomography. The results show that minute amounts (0.005 vol%) of highly dispersed carbon nanoparticles shift the mechanical properties to higher ductility at the expense of tensile strength. Despite changes in crystallinity due to nano-additives, the crystalline phases of polyamide 12 are retained. Layer bonding and part densities strongly depend on the quantity and dispersion quality of the nanoparticles. Nanoparticle loadings for CO_2_ laser-operated PBF show only minor changes in material properties, while the potential is greater at lower laser wavelengths.

## 1. Introduction

For over 30 years, Additive Manufacturing (AM) has been known for its ability to produce customized parts of high complexity and resolution [1]. Next to the AM of inorganic materials [2,3], laser-based powder bed fusion of organic polymers (LB-PBF-P, according to ISO/ASTM 52911-2:2019) has experienced increased interest in terms of research and development over the last decade [4]. However, AM still requires a lot of operator know-how as many external and internal variables influence the process, e.g., feedstock material [5] or build orientation [6]. In this regard, the interplay of parameters related to the laser source, such as laser power, laser beam diameter, laser scanning speed, and hatch distance, with machine-related parameters, such as powder layer height, process temperature, and recoating speed, is crucial for the successful manufacture of dimensionally accurate three-dimensional parts. The most common way to combine the most important process parameters is the volume energy density [4,7].

Next to the process parameters, first and foremost, bulk solid properties affect the packing density, the flowability and, thus, the spreadability during the AM process [8,9]. If the adaptation of process parameters reaches its limits, the additivation of the base polymer powder with nanomaterials provides a promising tool to steer the material properties in a certain direction or to add new functionalities, e.g., electrical conductivity or magnetism [4,10]. The most common examples of nano-additives for PA12 are carbon-based materials due to their vast availability and attractive properties [4,11,12]. Interestingly, the results of studies on carbon additivation reported in the literature differ significantly for LB-PBF-P. On the one hand, the addition of carbon nanomaterials led to a degradation of mechanical properties under the same process conditions [13,14,15], while, on the other hand, major improvements were reported [16,17,18,19]. However, these improvements mainly correlate with the anisotropic properties of the nanofillers, e.g., carbon nanotubes and fibers, or the optimization of process strategies to improve the processability and the densification of final parts. In this context, a good dispersion of the nanomaterial on the polymer particle is essential to avoid heat accumulation at agglomerate positions and, thus, impairment of mechanical properties [4,15,18]. The quality of the dispersion depends on many influencing factors, for example, the chemical nature of the nano-additives [20], their dosage [21,22], the additivation method [23], and the preparation method of the nanocomposites [15].

While studies have already discussed the importance of a good dispersion for nano-additives on polymer particles [24], a comparative evaluation between different dispersion qualities is still missing, especially for LB-PBF of polymer composites with nanoparticle quantities below 0.1 vol%. Our study closes this gap by processing PA12 powder composites modified with carbon nanoparticles (CNP) [23] into three-dimensional specimens to analyze the influence of two additivation methods of different dispersion qualities on the process and material behavior. In order to gain an initial insight into the influence of another nanoparticle group on the LB-PBF-P process and the mechanical part properties, PA12 specimens with 0.05 vol% colloidally additivated silver nanoparticles (Ag-NP) [20] were manufactured and mechanically tested analogously to PA12/CNP. The results of this study provide a deeper understanding of the importance of the dispersion quality of minute amounts of nanoparticles and its impact on the LB-PBF-P process and the part qualities.

## 2. Materials and Methods

### 2.1. Nano-Additivation Process

The adhesion of CNP (CARBON BLACK, Orion Engineered Carbons) onto PA12 powder (EVONIK VESTOSINT 1115, Evonik Industries, Essen, Germany) was achieved by dry coating (DC) and colloidal additivation (Coll) [23,25]. The polymer powder particles are potato-shaped due to their formation through precipitation from ethanol under pressure [26]. The influence of nano-additivation on the polymer shapes is discussed in-depth in [27]. For dry coating, the polymer powder was mixed with CNP powder in a rotating drum for 2 h and then sifted with a 125 µm sieve. For the colloidal deposition process, CNP powder was dispersed in deionized water (washed) by ultrasonic treatment (50 mg/L), followed by laser irradiation with a 10 ps-laser at a wavelength of 532 nm (Edgewave PX400-3-GH, Würselen, Germany, 80 kHz, 30 W, 150 mJ/cm^2^, 375 µJ/pulse, 0.25 mm^2^ spot size) in a liquid jet setup [25] and finally mixed as a colloid with an aqueous PA12 suspension (50 g/L) [28]. Finally, the colloidal suspension was stirred for 5 min, filtered, dried (24 h at 50 °C), and sifted using a 125 µm sieve. Silver nanoparticles were only added colloidally to PA12 [29,30] for an initial comparison between organic and inorganic nanomaterials, and are discussed in more detail in [20,31].

### 2.2. Polymer Powder Characterization

Since the size and shape of polymer powder particles have a significant influence on the structure and properties of manufactured parts [32], the different PA12 powder compositions with CNP and Ag-NP were analyzed and characterized by measuring the Hausner ratio, utilizing dynamic image analysis (Camsizer X2, Microtrac Retsch, Haan, Germany) and a ring shear tester (RST-XS, Dietmar Schulze Schüttgutmesstechnik, Wolfenbüttel, Germany) at a pre-consolidation stress of 1 kPa within a small ring shear cell (V = 31 cm^3^) [20,23]. An evaluation of the flowability with the Hausner ratio is limited due to its low methodological sensitivity and lack of transferability to the powder application procedure in LB-PBF-P [27]. Therefore, ring shear tests deliver more reliable results, as this method is more sensitive to small changes in powder composition [33,34]. Additional measurements of PA12/CNP and PA12/Ag were performed three times to complete the powder characteristics under the same conditions as previous studies [20,23]. Since only the colloidal additivation was accompanied by a preceding washing step of PA12 powder, comparisons were made with washed and as-received PA12 powder, accordingly. Two-sample *t*-tests were performed to assess the statistical significance.

### 2.3. Thermal Analysis by Differential Scanning Calorimetry (DSC)

Previous studies analyzed the pure PA12 powder and the additivated powder with CNP and Ag-NP by dynamic DSC (DSC 822e, Mettler Toledo, Columbus, OH, USA) [20,23]. This way, the process temperatures for LB-PBF-P were estimated. After processing the powders in LB-PBF-P (see Section 2.4), cut-up pieces from the center of the manufactured square plates of the different PA12 powder compositions were heated from 25 °C to 230 °C at a rate of 10 K/min to determine the melting temperatures, enthalpies, and crystallinities. The crystallinity $X_{c}$ of the processed samples was calculated according to Equation (1) [35,36]:

(1) $$X_{c}=\frac{\Delta H_{m}}{\Delta H_{100}\cdot \left(1-w_{f}\right)}=\frac{\Delta H_{m}}{209.3\frac{J}{g}\cdot \left(1-w_{f}\right)}$$

the heat of fusion of the sample $\Delta H_{m}$ was extracted from the measurements; the heat of fusion $\Delta H_{100}$ of 100% crystalline PA12 is found in the literature [37]. The parameter $w_{f}$ gives the weight percentage of nanoparticles in the composite. Each sample was analyzed three times, leading to a total of 18 runs. The evaluation of the results was performed with the Mettler Toledo STARe Evaluation Software 16.10 (Columbus, OH, USA). The integral tangential baseline was used for the calculation of the relevant enthalpies. Two-sample *t*-tests were performed to include the statistical significance.

### 2.4. Laser Powder Bed Fusion of Polymers (LB-PBF-P)

Process development of powder bed fusion was conducted with a CO_2_ laser-based LB-PBF-P machine (Eosint P385, EOS, Krailling, Germany) of 0.6 mm beam diameter at the working plane. The coater speed was set to 50 mm/s to ensure a smooth and homogeneous powder surface. Pure PA12 powder was used as a base layer of 10 mm height for thermal decoupling from the building platform. To process small amounts of powder, the process chamber was downsized via a reduction in the coater length from 350 mm to 100 mm. In order to avoid premature melting of the top layer of the PA12/CNP powders due to the change in emissivity, the process temperature was lowered successively until coalescence was avoided. Conversely, the PA12/Ag powders needed to be processed at higher temperatures. Furthermore, the power of the IR-emitters was set to the same power range in the machine software for processing of pure PA12 in order to provide similar thermal conditions for every material composition. A powder layer of 0.6 mm was added to the last molten areas to reduce warpage during the cooling phase. Five tensile 1BA specimens (DIN EN ISO 527-2) of 2 mm thickness and five square plates (15 × 15 × 2 mm^3^) were produced in the x–y-plane with a layer height of 100 µm for each material composition. An alternating hatching was chosen for every layer between the x and y directions. The aim was to find a set of process parameters that could be used to process every material composition under the same conditions instead of optimizing the process parameters towards the best processability, density, or mechanical properties for individual material composites. In this way, the influence of nanoparticles on the material behavior and the material limitations of the composites could be examined and evaluated. However, the same energy density could not be used for 0.05 vol% CNP. Lowering the energy density of the reference material of pure PA12 was not an option as it led to a lack of layer bonding. Thus, the laser power was lowered successively to such an extent that the thermal difference between the material temperature and the laser energy input was reduced, and curling no longer occurred. To evaluate the performance of the integrated CO_2_ laser source, laser power measurements were conducted with a laser power meter (LM-200, Coherent, Santa Clara, CA, USA). In the onboard software of the LB-PBF machine, the percentage of the laser power was set in 1% steps from 5% to 10% and in 5% steps from 10% to 100% laser power. In doing so, the nominal and real output power values could be extracted for the experiments (Figure S1). Table S1 summarizes the process parameters and resulting energy densities for the different powder composite materials. At the end of the building process, the IR-emitters were switched off. The pyrometer data of the LB-PBF machine showed that the powder bed temperature dropped from the process temperature to 120 °C, which is below the endset temperature of crystallization, at a declining cooling rate of approximately 15 K/min to 1 K/min. The process chamber was allowed to further cool down and remained closed for at least 12 h.

### 2.5. Dimensional Accuracy and Tensile Testing of Specimens

The thickness and the width of the measuring range of the tensile bars were measured with a micrometer with an accuracy of 0.01 mm. Tensile tests were performed according to DIN EN ISO 527 on the universal testing machine (Quasar 100, Cesare Galdabini, Vigevano, Italy) at room temperature with a load cell of 10 kN and a contact extensometer. The Young’s modulus was measured at a speed of 0.5 mm/min until an elongation of 0.3% was reached, followed by a speed of 20 mm/min to measure the ultimate tensile strength and the ultimate elongation.

### 2.6. Polarized Light and Scanning Electron Microscopy (SEM)

The square LB-PBF specimens were cut with a microtome (Leica Biosystems, Wetzlar, Germany) to 10 µm slices and placed on microscope slides with immersion oil. A light microscope (Metalloplan, Leitz/Leica, Wetzlar, Germany) with two polarizers was used to analyze the layer bonding and the birefringence of the crystalline structures. The surfaces and tensile fractured surfaces of PBF specimens were imaged using a scanning electron microscope (GeminiSEM 500, Zeiss, Oberkochen, Germany) equipped with an SE2 detector, an acceleration voltage of 1 kV, and an aperture of 15 µm. Images were taken of the top surface, as well as the edges and centers of the fractured surfaces of the tensile specimens.

### 2.7. X-ray Diffraction (XRD) and Infrared (IR) Spectroscopy

X-ray diffraction (Empyrean series 2, Malvern Panalytical, Worcestershire, UK) using Cu K_α_ radiation (154 pm, 40 kV, 40 mA) was performed with an Empyrean diffractometer (Panalytical) in Bragg–Brentano geometry. The incidence beam optics comprised the Bragg–Brentano-HD module, fixed divergence (1/8°), anti-scatter (1/8°) slits, and 0.04 rad Soller slits. The diffracted beam optics comprised Soller slits (0.04 rad), an anti-scatter slit (16.8 mm), and a GaliPIX 3D detector (Panalytical). Sample specimens were fixed in the sample holder and rotated during measurement. The diffractograms were collected in the range of 5° ≤ 2θ ≤ 90° at a step size of 0.014° and a measuring time of 25 s per step. Phase analysis and Rietveld refinements of the obtained diffraction patterns were performed with Profex 4.3 (Solothurn, Switzerland), a GUI of the BGMN Rietveld Analysis Program (Dresden, Germany), to determine the phase composition and the unit cell parameters, but also to quantify the crystallite sizes. Infrared (IR) spectra of the specimens were recorded in attenuated total reflection (ATR) geometry in the spectral range from 6000 cm^−1^ to 400 cm^−1^ at a resolution of 2 cm^−1^ using a Fourier-transform infrared (FT-IR) spectrometer (FTS3100, Digilab, Hopkinton, MA, USA). The spectra were normalized to their respective maximum absorbance. Baseline corrections were performed manually. Pure PA12 and PA12/CNP specimens were analyzed this way.

### 2.8. Micro-Computed Tomography (µ-CT)

The specimens of PA12 and PA12/CNP were examined by X-ray microtomography to determine the process-induced porosity and the pores’ sphericity. This enabled the analysis of pore size and morphology in the micrometer range and the statistical evaluation of the pore characteristics. The type XT H 160 μ-CT system (Nikon, Tokyo, Japan), equipped with a microfocus X-ray source (tungsten) with a maximum voltage of 160 kV and a 3 µm threshold for the 3D scan, was used for the investigations. The 2D images were reconstructed into a 3D image using the CT Pro 3D software (Nikon, Tokyo, Japan). Subsequently, it was loaded into the analyzing and visualizing software VGStudio Max 2.2 (Volume Graphics, Heidelberg, Germany). The corresponding scanning parameters can be seen in Table 1.

After the scan, the images were reconstructed and loaded into the VGStudio Max 2.2 analysis and visualization software (Volume Graphics GmbH, Heidelberg, Germany). Afterwards, algorithm-specific voxels were characterized with the “VGDefX (v2.2)” as defects based on their grey value compared to a defined local threshold for contrast. The theoretical resolution of the system is 3 µm; however, due to the dimensions of the specimens, the minimum accurate detectable pore size increased to 15 µm. By setting the minimum pore size in the defect analysis program according to the effective pixel size of the specimens (15 µm) and checking the detected probability of the detected pores, the pores could be distinguished from the noise.

## 3. Results and Discussion

### 3.1. Material Characterization of Polymer Powder Composites

**Figure 1:**
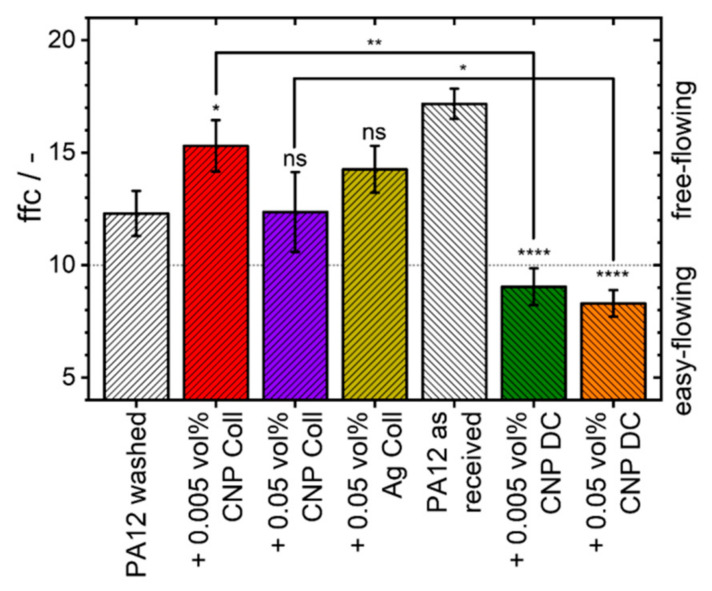
Flowability characteristics of PA12 powder and its composites measured by a ring shear cell at a pre-consolidation stress of 1 kPa. Due to the additional washing step before the colloidal additivation, colloidal composite powders are compared to washed PA12 powder while dry-coated composites are compared to PA12 powder as received. An increase in significance is depicted with an increase in the number of asterisks, while no significant differences are declared as “ns” (p>0.05). Results are based on three measurements.

An overview of all measured powder characteristics can be found in Table S2. While the difference between the flowability values measured with the Hausner ratio is insignificant, the ring shear test results indicate distinguishable deviations. Figure 1 summarizes the flowability (ffc-value) results of the differently additivated PA12 powders.

For instance, 0.005 vol% of CNP significantly increases the flowability of PA12 powder (*; p≤0.05) when additivated with the colloidal approach, while dry coating significantly (****; p≤0.0001) lowers the free-flowing (ffc > 10) powder properties to easy-flowing (ffc < 10) [38]. An increase in the CNP dosage to 0.05 vol% does not impair the flowability by colloidal additivation, while dry coating further reduces the flowability of the powder. The poorer outcomes can be ascribed to the stronger mechanical forces during the dry coating process, which lead to an increased inter-particle cohesion due to fines [23,27]. Regardless of the additivation method, 0.05 vol% of CNP leads to poorer flowing powders than smaller quantities due to an increased amount of CNP agglomerates on the polymer particle surfaces [23]. In comparison, the colloidal additivation of the same amount of Ag-NP slightly increases (ns; p>0.05) the flowability. Based on these results, the colloidal additivation process is the more suitable option for the additivation of nanoparticles since the good flowability of the base powder material is maintained.

### 3.2. Thermal Evaluation by Differential Scanning Calorimetry (DSC)

**Figure 2:**
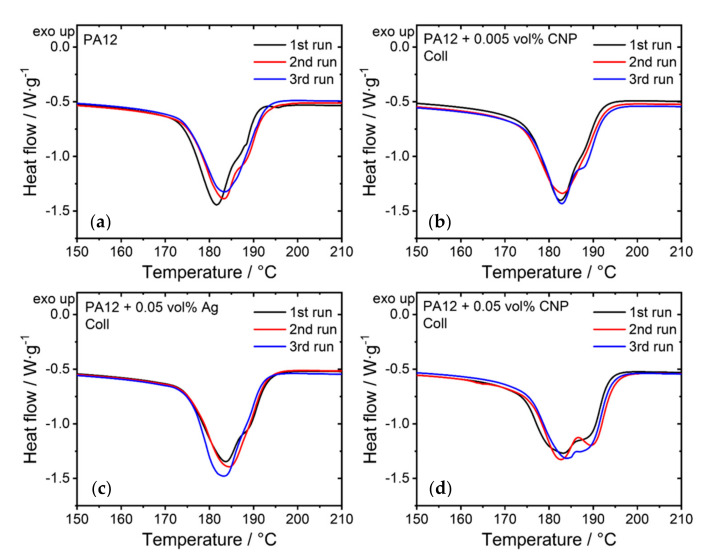
Three heating curves of the LB-PBF-P specimens made of (**a**) pure PA12, (**b**) PA12 and 0.005 vol% carbon nanoparticles, (**c**) PA12 and 0.05 vol% silver nanoparticles, and (**d**) PA12 and 0.05 vol% carbon nanoparticles. The addition of the nanoparticles was performed via colloidal additivation. The heating rate was 10 K/min.

Evaluating the melting behavior of the PA12 powders during the heating stage of the DSC helps to estimate the processing temperature for LB-PBF-P. Regardless of the nano-additives, each powder composition exhibits an endothermal increase in heat flow at around 170 °C during the heating phase. This value sets the starting point for the LB-PBF-P experiments. The processed samples show a second peak around 190 °C, which correlates with the higher melting temperatures of unmolten particles [39]. This peak becomes more pronounced as more CNP nanoparticles are added (Figure 2), which means that the number of unmelted particles increases with the dosage of nanoparticles.

Consequently, it provides an initial indication of the poorer expected material properties of the composites compared to pure PA12 due to reduced part density and layer bonding. However, the additions of 0.005 vol% CNP and 0.05 vol% Ag-NP retain the curve characteristics of pure PA12. The exact dimensions of the second peak of every material were not averaged due to large deviations between the three runs of each composition (Figure 2). Thus, the quantitative difference between the material compositions and the additivation methods is insignificant.

**Figure 3:**
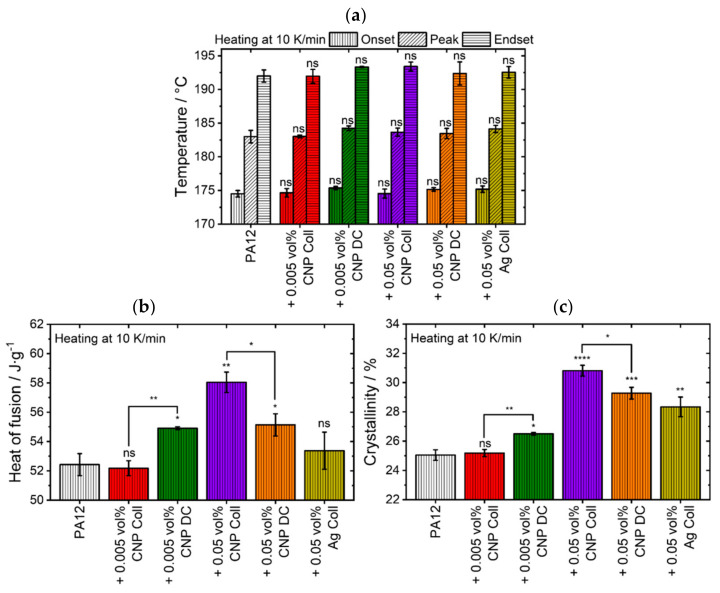
Averaged thermal values of different material compositions showing their results of (**a**) peak melting temperature values, (**b**) heat of fusion, and (**c**) crystallinity. The level of significance increases with the number of asterisks, while “ns” stands for an insignificant difference.

Since the second peak does not change the main peak positions, the temperature onset, peak, and endset do not change significantly (ns; p>0.05) by any additivation (Figure 3a). Yet, the overall crystallinity increases significantly for all additivated samples except for PA12 colloidally additivated with 0.005 vol% CNP (Figure 3c) due to the broadening of the second peak. The differences between the two additivation methods are more prominent for 0.005 vol% CNP (**; p≤0.01) than for 0.05 vol% CNP (*; p≤0.05). Therefore, an increase in crystallinity is not an initial indication of improved mechanical properties since the higher crystallinity values may come from an increased number of unmelted, but highly crystalline, polymer particles. Moreover, the results of the heat of fusion and crystallinity do not provide information about layer bonding. If an increase in crystallinity by nanoparticles has macroscopic advantages for the properties of the mechanical part, further analytic examinations are required. For instance, microscopic investigations need to be conducted to evaluate the connection between subsequent layers and the crystal growth behavior at these interfaces.

### 3.3. LB-PBF of PA12 and Composites

Differences between colloidal additivation and dry coating were noticeable during powder recoating, where the colloidally additivated powder could be spread more homogeneously than the dry-coated powder. During processing, specimens of colloidally additivated PA12 powder of 0.05 vol% CNP required less energy (0.115 J/mm^3^) to be built compared to the dry-coated variant (0.127 J/mm^3^). The temperature had to be decreased from 171 °C to 169 °C when increasing the CNP amount to 0.05 vol%. Otherwise, overly high temperatures and energy densities lead to a displacement of the specimens’ molten layers, ultimately resulting in a build job failure. Thus, lower temperatures and less energy were required to process and bond the polymer layers with the addition of minute amounts of CNP. By contrast, PA12 with 0.05 vol% Ag could be processed without any curling after raising the temperature by one degree Celsius while maintaining the same process parameters and energy density as pure PA12. When qualitatively comparing the spreadability of CNP and Ag-NP at 0.05 vol% during the recoating process, the powder bed of additivated PA12 powders with Ag-NP appeared more homogeneously distributed than the CNP counterpart. At a lower dose of 0.005 vol%, the dry-coated CNP powders exhibited faster curling after powder application than the colloidally additivated ones. It was found that the reduced process window of CNP additivated powders [23] leads to faster warpage of specimens during the cooling phase.

### 3.4. Microscopic Evaluation of LB-PBF-P Samples

**Figure 4:**
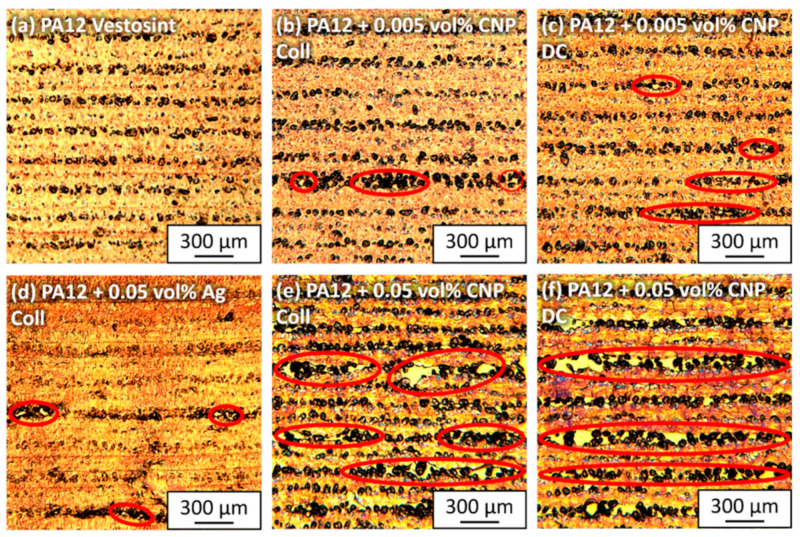
Microscopic polarized images of 10 µm sliced LB-PBF-P specimens made of (**a**) pure PA12, (**b**,**c**) PA12 and 0.005 vol% carbon nanoparticles, (**d**) PA12 and 0.05 vol% silver nanoparticles, and (**e**,**f**) PA12 and 0.05 vol% carbon nanoparticles. PA12 powders were additivated (**b**,**d**,**e**) with the colloidal deposition and (**c**,**f**) with the dry coating method. The images provide an overview of the processed layers in a horizontal position, where higher amounts of carbon nanoparticles lead to poorer layer bonding.

Polarization images at low magnification (Figure 4) reveal the quality of the layer bonds of each processed material composition. The presented images show horizontal layers built in the z-direction. Pure PA12 shows consistent bonding of individual layers (Figure 4a) with some unmelted particles, which is a typical phenomenon for LB-PBF-P results. The degree of particle melt could be increased by higher laser energy inputs, but the aim of this study was to evaluate the influence of nanoparticles on the processability and material properties. The first indications of a reduction in particle melting became apparent when adding 0.005 vol% CNP to the surfaces of the PA12 particles. Even though the process temperature remained the same, layer bonding was partially disrupted by an increased number of agglomerated unmelted particles (Figure 4b,c). Visual differences between the colloidal and dry-coated additivation procedure were non-existent at this low volume of CNP.

When increasing the dosage of CNP to 0.05 vol%, the laser power had to be lowered by 15% for dry-coated powders and 24% for colloidally additivated powders (Table S1) to process 20 layers without build failures. However, this led to a deterioration in bonding quality between the layers. Although the dry-coated specimens absorbed an energy of 0.127 J/mm^3^, their microscopic results show larger and longer gaps between the layers (Figure 4e) than the colloidal additivated specimens (Figure 4f), which received a lower laser energy of 0.115 J/mm^3^. More agglomerated CNP from dry coating are most likely the cause of this outcome. By comparison, good layer bonding is given for the same amount of Ag-NP (Figure 4d) with occasional smaller gaps. Reasons for the differences between CNP and Ag-NP are either due to the energy distribution, which is redirected by the absorptive and emissive properties of the CNP, or the polymer–polymer connection, which is interrupted by nanoparticle interactions of opposite layers. Another reason could be the difference in the Hamaker constant between CNP and Ag-NP [40,41]. In addition, the change of bonding behavior between the polymer and the organic CNP or inorganic Ag-NP could be the cause.

**Figure 5:**
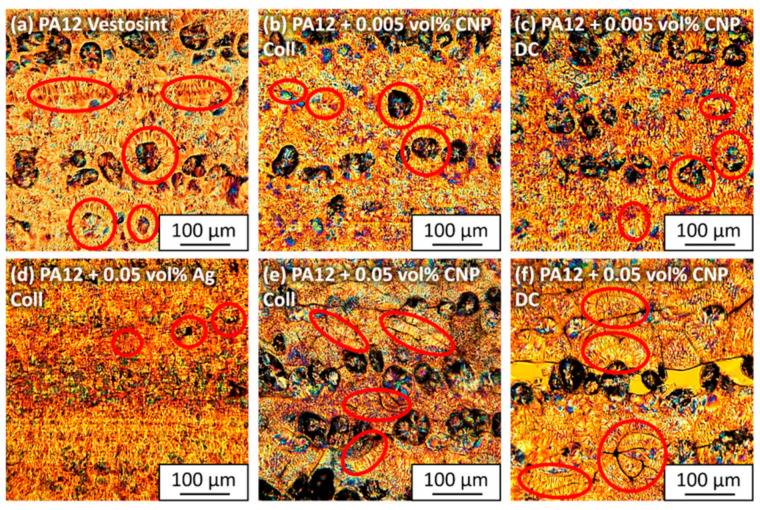
Magnified microscopic polarized images of 10 µm sliced LB-PBF-P specimens made of (**a**) pure PA12, (**b**,**c**) PA12 and 0.005 vol% carbon nanoparticles, (**d**) PA12 and 0.05 vol% silver nanoparticles, and (**e**,**f**) PA12 and 0.05 vol% carbon nanoparticles. PA12 powders were additivated (**b**,**d**,**e**) with the colloidal deposition and (**c**,**f**) with the dry coating method. The images provide a more detailed view of the developed crystalline structures and the positions of carbon and silver nanoparticles in the cooled polymer melt.

With microscopy images of higher magnification (Figure 5), lamellar structures can be identified. The typical crystalline structures of PA12 with lamellae up to 25 µm are visible, originating from unmelted particle cores or random impurities (Figure 5a). The dimensions of these lamellae, which develop during the cooling phase of LB-PBF, correlate well with the microscopic results of the calorimetric powder measurements [23] since their lengths lie between the values of the cooling rates at 0.5 K/min and 20 K/min. Spherical and ellipsoidal structures can be found, as observed in previous studies [23,25]. Adding 0.005 vol% CNP does not increase the frequency of crystalline structures, but seemingly reduces them. There are no apparent differences between the two additivation methods at this low dose of nanoparticles (Figure 5b,c). However, formations of agglomerated nanoparticles to chain-like structures (Figure 5e,f) can be identified at 0.05 vol% CNP. Even though the nanoparticles occasionally induce lamellar growth across layer boundaries, they also introduce new interfaces that predetermine mechanical weak points. While there are larger CNP clusters in the dry-coated specimens, the colloidal additivation leads to a more homogeneous distribution of the CNP in the melt. It is expected that dry-coated samples will have poorer mechanical properties due to the higher frequency of clusters. The lamellar structures cannot be identified in the PA12 sample with 0.05 vol% Ag-NP (Figure 5d). Only crystalline growth from unmelted particles can be found. As expected from the thermal powder analyses [20], no nucleation effects of the Ag-NP can be detected by microscopic evaluations. However, both microscopic and DSC evaluations are limited as they only represent a small percentage of the total sample. Three-dimensional analysis methods, such as µ-CT (see Section 3.8), can help to better understand the internal structures of the entire sample.

Our previous hypothesis [23] that CNP can introduce a certain anisotropy into the material system, which can be beneficial for increasing the layer bonding, cannot be confirmed. Instead, a CNP dose of 0.05 vol% is high enough to compromise layer bonding if the process parameters are not changed. However, lower quantities can be used to tailor mechanical properties.

### 3.5. Dimensions and Mechanical Properties of Tensile Bar Specimens

**Figure 6:**
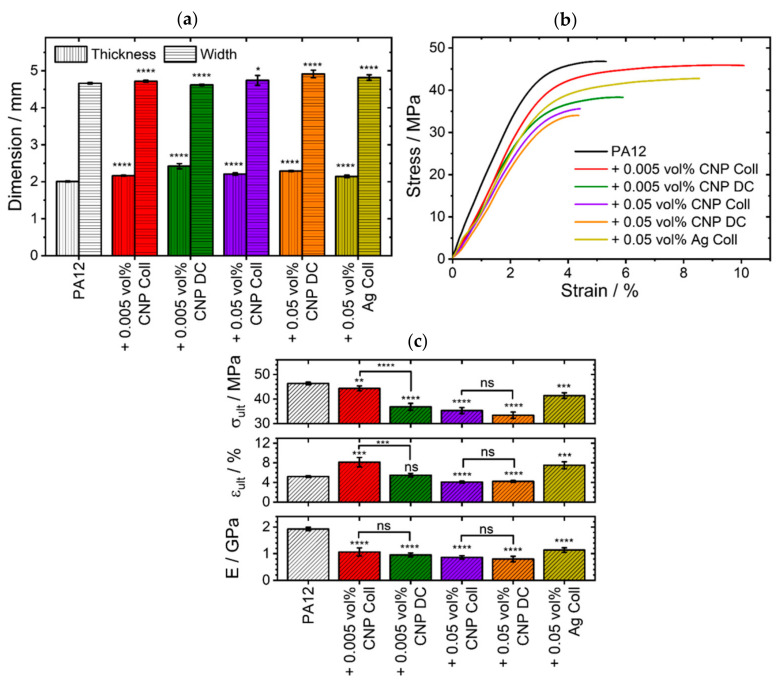
The overview of the tensile test results shows the LB-PBF-P specimens of different material compositions with regard to their (**a**) measured dimensions, (**b**) exemplary stress–strain curves, and (**c**) mechanical properties of the ultimate tensile strength ($\sigma_{ult}$), ultimate elongation ($\varepsilon_{ult}$ ) and Young’s modulus (E ). The level of significance increases with the number of asterisks, while “ns” stands for an insignificant difference.

The dimensions of the reference specimens of pure PA12 were 2.01±0.02 mm in thickness and 4.66±0.02 mm in width (Figure 6a). The specimens exhibit a Young’s modulus E of 1.93±0.07 GPa, an ultimate tensile strength $\sigma_{ult}$ of 46.4±0.5 MPa, and an ultimate elongation $\varepsilon_{ult}$ of 5.2±0.2% (Figure 6b,c). These values are well within the typical range of mechanical properties of specimens produced by LB-PBF-P [4]. The exact target width of 5 mm was not achieved since no contour exposure was used in this study. However, since the values are within the range of the DIN standard and show only a small spread, the process parameters still provide a good reproducibility for pure PA12 powders in LB-PBF.

The low deviations among the five specimens prove the consistently good processability of the powder composites (Figure 6), especially at low doses of nanoparticles (<0.05 vol%). However, the deviations increase at higher nanoparticle concentrations, which corresponds to the poorer processabilities of these powder composites. Further differences are caused by the additivation methods. When comparing the dry-coated PA12 containing 0.005 vol% CNP with pure PA12 (Figure 6a), the width of the dry-coated specimens is significantly (0.9%) (****; p≤0.0001) lower, while the thickness values are significantly (20.7%) (****; p≤0.0001) higher. Faster warpage of dry-coated specimens with 0.005 vol% CNP during cooling lead to the specimens’ greater thickness and narrower width. This results from the reduced processing window due to an increase in crystallization temperatures induced by the nucleation effect of CNP [23]. By comparison, the colloidally additivated specimens of the same CNP concentration exhibit a significant (****; p≤0.0001) increase in thickness and width by only 1.2% and 7.8%, respectively. Since there are no significant thermal differences between these two 0.005 vol% PA12/CNP powders, the main reason should be ascribed to the quality of the nanoparticle deposition and an increased expected agglomeration of nanoparticles in the melt. Overall, the specimens built from composites show significantly higher dimensional values than those from pure PA12. This correlates with the unintentional melting and coalescence of neighboring particles induced by the CNP (see Section 3.6).

The difference between the two additivation methods at minute amounts of 0.005 vol% CNP can also be recognized in the tensile results (Figure 6b,c). Here, dry-coated specimens exhibit a $\sigma_{ult}$ that is significantly (20.6%) (****; p≤0.0001) lower than pure PA12, and a $\varepsilon_{ult}$, which increases insignificantly (5.4%) (ns; p>0.05). In comparison, the colloidal additivation of the same quantity of CNP also decreases $\sigma_{ult}$ significantly (**; p≤0.01), but only by 4.4%, while $\varepsilon_{ult}$ is increased significantly (***; p≤0.001) by 56.5%. While the difference between the dimensional accuracy (Figure 6a) and the mechanical properties (Figure 6b,c) diminishes when increasing the volume of CNP to 0.05 vol%, there are small but insignificant (ns; p>0.05) differences between colloidal additivation and dry coating. For instance, the width increases significantly (****; p≤0.0001), by 5.4%, and the thickness increases significantly (****; p≤0.0001), by 14.1%, for dry-coated specimens. On the other hand, the dimensions of the colloidally additivated specimens have a smaller, but significant (*; p≤0.05), growth of 1.7% and 10.2% (****; p≤0.0001). Similarly, every measurand of the mechanical properties is significantly (****; p≤0.0001) deteriorated at 0.05 vol% of CNP. Interestingly, the colloidal additivation showed a 4.9% higher tensile strength and a 3.3% higher Young’s modulus than the dry-coated specimens, even though the colloidally additivated powder received 9.4% less energy during the LB-PBF-P process (Table S1). However, this difference between the two additivation methods is insignificant (ns; p>0.05). When changing the nano-additives to 0.05 vol% silver, the dimensions increase significantly (****; p≤0.0001) by 3.3% in width and 6.9% in thickness. At the same time, the mechanical properties are close to values of 0.005 vol% CNP by colloidal additivation. Finally, all composites have a significantly (****; p≤0.0001) lower Young’s modulus than pure PA12 (Figure 6c). The reasons for the decrease in tensile strength and Young’s modulus, but the increase in elongation, could be due to a weakened interaction between the nanofiller and the polymer matrix (e.g., low crosslink density) [13]. A low dispersion and, thus, a higher probability of agglomeration of nanoparticles would explain the overall impairment of dimensions and mechanical properties by dry coating of 0.005 vol% CNP. Conversely, the chances of nanoparticles agglomerating increase at higher quantities of CNP, limiting the possibility of a better dispersion to induce macroscopic changes. Other nucleating nanomaterials should be chosen instead of CNP to improve the interaction between the nano-additives and the polymer matrix.

In general, we could show that small volume fractions of CNP and Ag-NP are already enough to significantly influence the dimensional and mechanical properties of LB-PBF specimens. The quality of the dispersion plays a decisive role in this. The impact of CNP on the dimensional and mechanical properties is greater than that of the same dose of Ag-NP without nucleation properties. However, for CO_2_ laser-operated powder bed fusion processes, the influence of nanoparticles on the material behavior is limited and usually does not contribute to better mechanical properties. In this case, a compromise has to be made between introducing new material properties through nanoparticles (e.g., plasmonic [31,42] or magnetic properties [10]) and the mechanical properties of additively manufactured parts. However, if the wavelength of the laser source is in the near-infrared or visual wavelength range, absorption-enhancing nanoparticles of high dispersion become inevitable to effectively process polymer powders at these wavelengths [31].

### 3.6. Tensile Fractography

**Figure 7:**
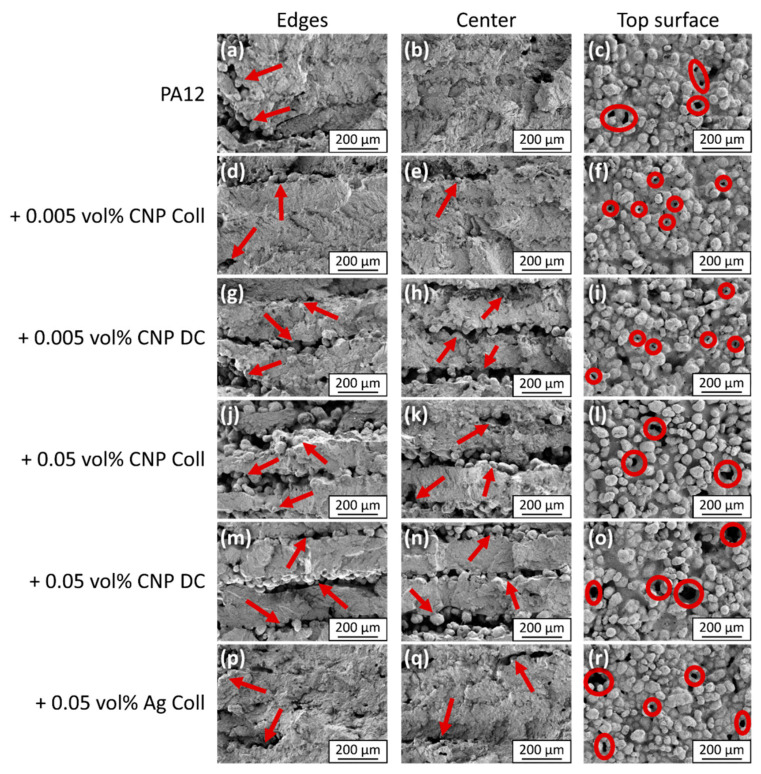
Collection of scanning electron microscopy images of the fractured surfaces of the tensile bars and of the top surface of the specimens. The left column (**a**,**d**,**g**,**j**,**m**,**p**) depicts the edges, and the middle column (**b**,**e**,**h**,**k**,**n**,**q**) shows the center of the fractured surface. The condition of the specimens’ top surface can be seen in the right column (**c**,**f**,**i**,**l**,**o**,**r**). The quantity of nanoparticles increases from top to bottom. Exemplarily, the red arrows mark unmelted polymer particles, while the red circles highlight voids.

Images of the top surface of the specimens help to evaluate the quality of the molten state of the outer layers, while images of the fractured surfaces are used to identify the fracture behavior under the influence of different amounts of nanoparticles. The presented images depict the processed horizontal layers perpendicular to the build direction (Figure 7).

**Figure 8:**
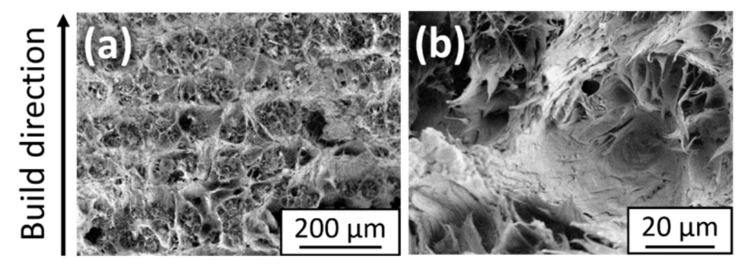
(**a**) Scanning electron microscopy images of the ductile area of fractured surfaces of the PA12 specimens. A more detailed view of the spherical fibrillated structures responsible for the ductility can be seen in (**b**).

PA12 shows overall good layer bonding without visible layer boundaries in the body of the sample (Figure 7b), while some lack of bonding exists around the edges (Figure 7a). The reason for this is the absence of contour parameters in this study, which are usually used in LB-PBF-P to mitigate this phenomenon. The layer bonding and, thus, the mechanical properties can be further improved by increasing the degree of particle melt with higher laser energy densities. However, this usually sacrifices the parts’ resolution and dimensional accuracy due to unwanted sintering of adjacent loose powder particles [26]. The fracture surface images primarily reveal brittle regions with some ductile areas around the edges of the sample (Figure 8a), which are believed to be the origin of fracture during tensile testing [26]. Upon closer inspection, the ductile areas consist of mainly spherical fibrillated structures indicating broken particle cores (Figure 8b). The condition of the top surfaces (Figure 7c) is typical for processed PA12 powder with partially molten and unmolten particles from the surrounding powder due to bleeding of thermal energy [43]. However, this has no adverse effect on the dimensional accuracy of the specimens since the thickness of the final parts is at the target value of 2 mm (Figure 6a). The seamless bonding of the layers further supports the good dimensional accuracy of the PA12 specimens. Optionally, the surface quality could be improved by an additional post-processing step involving grinding or polishing [44].

Adding 0.005 vol% CNP by colloidal additivation leads to more distinct layer boundaries of partially unmelted particles near the edges and in the center of the specimens (Figure 7d,e), while interlayer bonding is still present. The same quantity of CNP, but additivated by dry coating, results in enlarged gaps between individual layers near the edge and inside of the specimens (Figure 7g,h). These interrupted transitions are due to partially or fully unmelted particles, which correlate with the second peak, at around 190 °C, observed in DSC (Figure 2). The frequency of ductile areas is the highest for PA12 with 0.005 vol% CNP by colloidal additivation compared to pure PA12 and other specimens. This can be ascribed to the increased ultimate elongation of the tensile specimens (Figure 6c). Due to the same amount of CNP being present, the reasons for these differences are either the quality of dispersion [23] or the change of powder flowability during additivation, which influence the processability of the composite powders. These apparent differences in fractured surfaces between the colloidal additivation and the dry coating of 0.005 vol% CNP are reflected in their dimensional accuracy (Figure 6a) and mechanical properties (Figure 6b,c). The higher number of gaps explains the significant increase of 20.7% in thickness for the dry-coated specimens. However, the top surface structures show no evident differences between the two additivation methods for the specimens additivated with 0.005 vol% CNP (Figure 7f,i) and are equivalent to the pure PA12 results. The difference in fracture behavior between the two additivation methods is undistinguishable when increasing the dosage of CNP to 0.05 vol% (Figure 7j,k,m,n). These findings coincide with the insignificant differences (ns; p>0.05) in mechanical properties between the two differently additivated specimens (Figure 6c). The number of unmelted particles between the layers increases, further impairing the mechanical properties of the specimens. The only noticeable difference in the top surface structure is a higher number of voids within the molten surface. These voids are more pronounced for the dry-coated specimens than for the colloidally additivated ones. This could be an indication of an increased amount of escaping gas or polymer chain scission.

By contrast, the same quantity of Ag-NP leads to very similar results as pure PA12 with regard to layer bonding and layer boundary conditions (Figure 7p,q). Since Ag-NP do not induce crystal growth, in contrast to CNP, under LB-PBF cooling conditions (Figure 5d) [20], they can maintain the good processability of pure PA12 powder. Finally, a dispersion of good quality is particularly important for nucleating nanoparticles below a dose of 0.05 vol% if a shift in mechanical properties is desired.

### 3.7. X-ray Diffraction (XRD) and Infrared (IR) Spectroscopy

Polyamide 12 is known to crystallize depending on the acting stresses, temperature, and pressure in different polymorphic forms. Four crystalline phases, namely the alpha (α), alpha’ (α’), gamma (γ) and gamma’ (γ’) phases, are known [26,45,46,47], with the gamma phase being the most stable form at ambient conditions and the alpha form observed for PA12 annealed at elevated pressures [48]. In addition, an intermediate α” form was observed prior to the transformation of γ PA12 to the γ’ polymorph by drawing [49], respectively, the (intermediate) crystallization of PA12 from the melt in the α’ form with a subsequent transition to the γ form while cooling to room temperature [50]. The γ’ phase can be produced by melt quenching [47]. Precipitated PA12 powders have been reported in the literature to frequently show an ‘intermediate’ structure between the alpha and gamma phase that is characterized by two distinct reflexes found at 2θ around 20.9° and 22.0° (Cu K_α_) [23,26,50].

**Figure 9:**
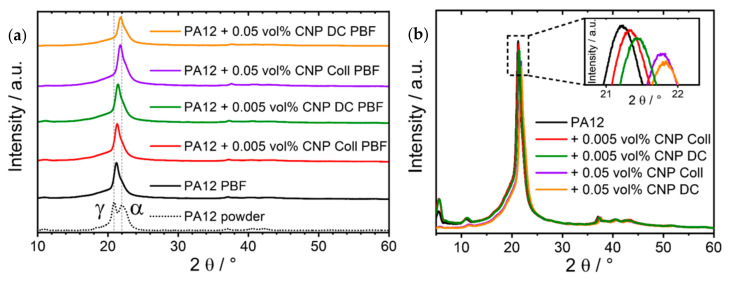
Diffraction patterns of PA12 powder and specimens of different compositions additivated by colloidal deposition and dry coating, depicted (**a**) as overviews separated from each other and (**b**) on top of each other with a zoomed-in picture of the shifted main reflex positions.

Diffraction patterns of the specimen produced from the different composite powders in comparison to PA12 powder are depicted in Figure 9 below. The experimental pattern could be described solely by the presence of a crystalline monoclinic (pseudohexagonal) γ PA12 phase when taking the structural data reported by Cojazzi et al. [51] and a polynomial background function into account, i.e., there are no indications of the presence of another crystalline PA12 phase. The specimens were produced by LB-PBF-P, i.e., the powder was first melted and then allowed to cool at relatively moderate cooling rates. At these conditions, i.e., the crystallization of PA12 from the melt, the γ form was previously reported to be formed [50]; however, also in LB-PBF-produced specimens, an intermediate structure between the α and γ phase was recently reported to be present in the precipitated feedstock [26]. Consequently, despite an apparent shift of the main reflex of less than 1° (Figure 9b), we can also confirm that under the chosen conditions, the CNP present in the composite powder do not trigger the nucleation of PA12 polymorphs other than γ in the built specimens. The unit cell parameters for the γ PA12 phase deduced by Rietveld refinement of the experimental diffraction pattern are summarized in Table 2. No trend indicating a dependence of unit-cell parameters or lattice spacings (Table S3) on any amount of NP present in the powder system could be deduced.

The crystallite sizes of the γ PA12 phase in the composite specimens were exemplarily determined from the refinement of the diffractograms for the (100), (002), and (020) orientations, characterized by 2θ angles of 21.4°, 21.9° and 5.5°, respectively (Table 3). No preferred crystallite orientation or texture can be deduced from the diffractograms. With increasing carbon black content, a slight increase in crystallite size in the (001) and (002) orientation can be noted, although this effect is relatively small.

According to Bain et al. [26] and Rhee and White [52], respectively, γ PA12 can be discriminated from α PA12 by the position of characteristic vibrations in the IR range, e.g., the Amide I (1635 cm^−1^ (α) vs. 1640 cm^−1^ (γ)) or the Amide II band (1540 cm^−1^ (α) vs. 1563 cm^−1^ (γ)). The band positions for amide I and amide II observed in ATR spectra of the samples collected at a resolution of 2 cm^−1^ are summarized in Table 4. Because of the instrumental resolution and the experimentally observed band positions, e.g., for the amide I between 1635 cm^−1^ and 1638 cm^−1^, respectively, representing very weak amide II bands, a clear assignment to one of the crystal phases or the deduction of the presence of a crystal mixture cannot be made from the IR spectra.

### 3.8. Micro-Computed Tomography (μ-CT)

**Figure 10:**
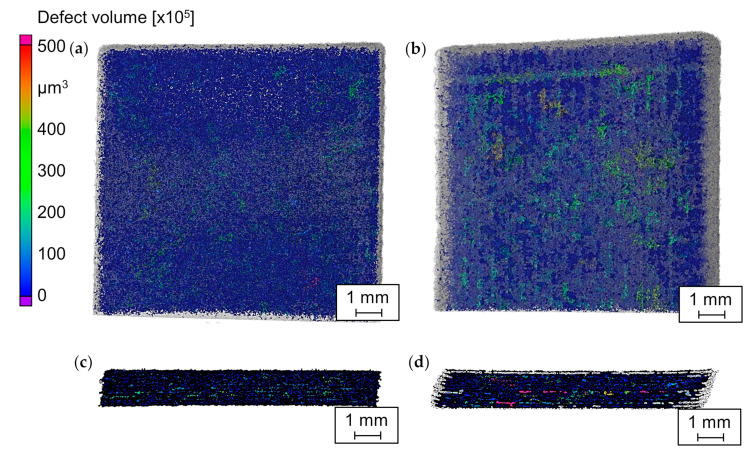
Results of µ-CT scans for specimens of pure PA12 in (**a**) top and (**c**) side view and of PA12 dry-coated with 0.05 vol% CNP in (**b**) top and (**d**) side view.

The effect of the additivation of CNP on the relative density of PA12 specimens is visible in Figure 10. Relatively large line-shaped defects in the PA12 specimens additivated with 0.05 vol% CNP and their layer-wise arrangement perpendicular to the building direction confirm the insufficient diffusion between the scanning layers. However, the defects are more distinguishable in the dry-coated specimens of 0.05 vol% CNP, where the lack of diffusion is more visible at the mid-layers of the specimens (Figure 10c,d).

**Figure 11:**
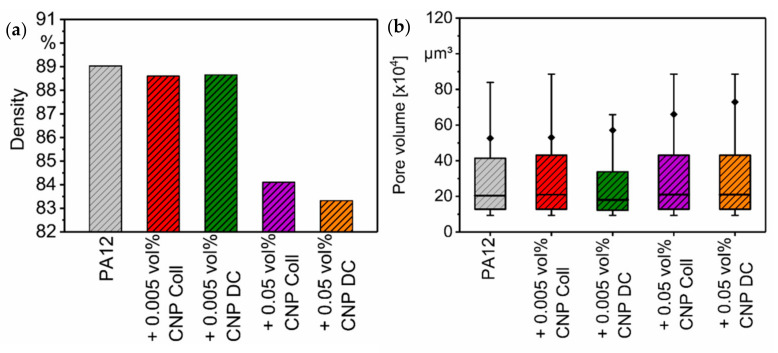
(**a**) Relative density and (**b**) pore size distribution of pure PA12 and PA12 specimens with CNP additivation.

It can be seen in Figure 11a that pure PA12 has the highest relative density of 89.0%. The addition of 0.005 vol% CNP reduces their relative density to 88.7% and 88.6% for the dry coating and the colloidal additivation methods, respectively. An increase in CNP to 0.05 vol% further decreases the relative density of the PA12 specimens to 83.3% and 84.1% for the dry coating and the colloidal additivation methods, respectively. This leaves comparable densities at 0.005 vol% CNP between dry coating and the colloidal additivation, while the colloidally additivated specimen shows a slightly higher density than the dry-coated counterpart. These results can be ascribed to the better layer bonding of lower quantities of CNP and the higher dispersion of nanoparticles by the colloidal additivation.

Figure 11b shows the size distribution, average pore size, and the pore counts within each specimen. In most of the specimens, pore volumes lay between 10 × 10^4^ µm^3^ and 85 × 10^4^ µm^3^. The average pore volume also increases as the quantity of CNP in the PA12 specimens increases. The colloidal additivation of 0.005 vol% CNP reduces the maximum pore size of PA12 from 85 × 10^4^ µm^3^ to 69 × 10^4^ µm^3^, while the dry coating method and higher amounts of CNP increase the maximum pore size by 5 × 10^4^ µm^3^. Therefore, lower quantities of CNP are less detrimental for the density of parts, in which case the dispersion of the nanoparticles plays a crucial role in the final part properties.

**Figure 12:**
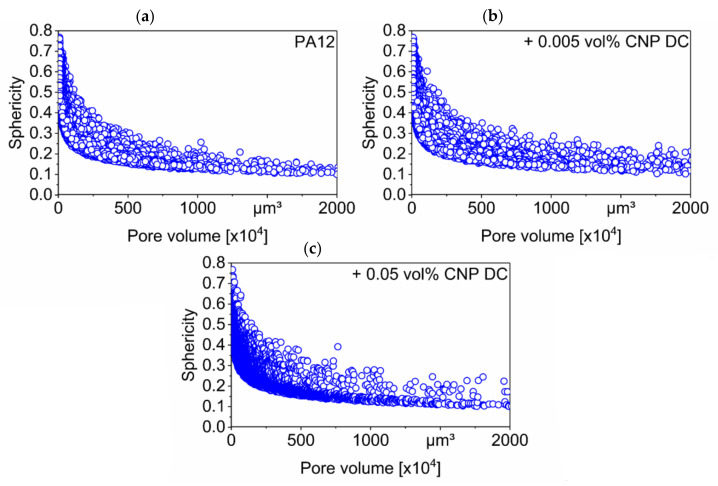
Sphericity measurements of the pores in specimens of (**a**) pure PA12, (**b**) PA12 dry-coated with 0.005 vol% CNP and (**c**) PA12 dry-coated with 0.05 vol% CNP.

Only very small pores in the specimens have high sphericity of up to approximately 0.8; however, it decreases drastically with the increase in the pore volume in all the specimens (Figure 12). As mentioned, large pores are long void spaces between the scan layers that are present due to a lack of fusion, which correlates with the increase in the lack of layer bonding when adding CNP to PA12 (Figure 7). As mentioned before, the process parameters were not chosen to achieve the highest density for every material composition but to investigate the influence of the nano-additivation on the material properties.

## 4. Conclusions

Well-dispersed nano-additives on polymer powders for laser-based powder bed fusion show potential for the tuning of material properties, but also pose challenges that must be overcome. In our study, we processed polyamide 12 powder that was modified with sub-monolayer quantities of carbon black nanoparticles, by means of two additivation methods of different dispersion qualities, to standardized tensile bars under the same process conditions. The two methods of polymer particle nano-coating were the aqueous colloidal deposition and the dry mechanical mixing. 

The differences between the two deposition methods become evident at different stages of the laser-based powder bed fusion process. During the evaluation of the powder flowability, only the colloidal additivation procedure keeps the free-flowing characteristic of pure polyamide 12 powder. This characteristic has an impact on the powder application of the powder bed fusion process, in which mechanically mixed powders lead to a less homogeneous powder bed. After processing the nanocomposites under same process conditions, 0.005 vol% of colloidally deposited carbon nanoparticles show an increase in the ductile material behavior of manufactured parts at the expense of tensile strength, while mechanically admixed nanoparticles reduce the mechanical properties of PA12. Despite increasing the crystallinity, carbon nanoparticles do not change the crystalline morphology of the intermediate form between the alpha and gamma phases of manufactured specimens. Higher amounts of nanoparticles than 0.005 vol% result in poorer layer bonding, reduced part densities with enlarged pores, and thus, worse mechanical properties, regardless of the deposition method. Ultimately, the quality of the dispersion of minute amounts of nanoparticles is critical to tailor the mechanical properties of thermoplastic parts by laser-based powder bed fusion. 

Future studies should include optimization of the laser-based powder bed fusion process of polymer nanocomposites towards high part densities of carbon nano-additivated specimens achieved through the adjustment of the powder bed temperature and volume energy density. Furthermore, lasers other than CO_2_ (in the near-infrared or visual wavelength range) should be used to facilitate an improved understanding of the influence of absorption-enhancing nanoparticles on the laser–material interaction.

## Figures and Tables

**Table 1:** Scanning parameters for the computed tomography scans (μ-CT).

Material	Beam Energy	Beam Current	Power	Effective Pixel Size	Exposure Rates
PA12/ PA12-CNP	99 kV	26 µA	2.5 W	15 µm	1.42 s, 0.707 fps

**Table 2:** Unit cell parameters for the monoclinic gamma PA12 phase as determined from Rietveld refinements of the diffraction pattern of processed specimens.

Material Composition	a in nm	b in nm	c in nm	β in °
PA12	0.4838	3.1810	0.9484	121.2
PA12 + 0.005 vol% CNP Coll	0.4838	3.1912	0.9513	120.8
PA12 + 0.05 vol% CNP Coll	0.4838	3.2219	0.9484	121.2
PA12 + 0.005 vol% CNP DC	0.4838	3.1854	0.9498	121.2
PA12 + 0.05 vol% CNP DC	0.4838	3.2213	0.9484	121.2

**Table 3:** Crystallite sizes in 100, 002, and 020 orientation for gamma PA12 in composite powder specimens as determined from Rietveld refinement.

Material Composition	Crystallite Size in nm			Error Crystallite Size in nm		
	(100)	(002)	(020)	(100)	(002)	(020)
PA12	8.21	7.12	6.478	0.15	0.14	0.099
PA12 + 0.005 vol% CNP Coll	8.53	6.58	6.426	0.16	0.14	0.041
PA12 + 0.05 vol% CNP Coll	9.43	8.66	5.503	0.19	0.11	0.057
PA12 + 0.005 vol% CNP DC	6.56	5.92	6.691	0.09	0.14	0.051
PA12 + 0.05 vol% CNP DC	9.29	9.51	5.037	0.20	0.14	0.055

**Table 4:** Positions of the amide I and amide II bands in PA12/CNP composite specimens.

Material Composition	Amide I in cm^−1^	Amide II in cm^−1^
PA12	1637	1543 and 1566
PA12 + 0.005 vol% CNP Coll	1637	1541
PA12 + 0.05 vol% CNP Coll	1635	1547 and 1566
PA12 + 0.005 vol% CNP DC	1638	-
PA12 + 0.05 vol% CNP DC	1636	1545

## Data Availability

The data presented in this study are available on request from the corresponding author. The data are not publicly available due to privacy.

## Supplementary Materials

The following are available online at https://www.mdpi.com/article/10.3390/ma14185322/s1, Figure S1: nominal and measured laser power, Table S1: process parameters for LB-PBF-P, Table S2: powder properties, Table S3: d-spacings (from XRD) of the manufactured specimens.
