# Supplementary material for: Quality over Quantity: How Different Dispersion Qualities of Minute Amounts of Nano-Additives Affect Material Properties in Powder Bed Fusion of Polyamide 12
Source: Materials (Basel). 2021 Sep 15;14(18):5322. doi: 10.3390/ma14185322 (PMC8465424; doi:10.3390/ma14185322)
Supplement: Supplementary file 1 [file materials-14-05322-s001.zip › materials-1322630-supplementary.pdf]

Supplementary information

Figure S1 shows the nominal and measured output laser power of the CO_2 laser from the EOSINT P385 LB-PBF-P machine.

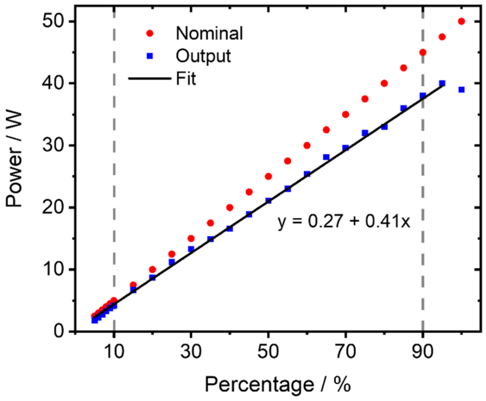

Figure S1. Nominal and measured laser power. Nominal and measured output laser power of LB-PBF-P machine EOSINT P385.

The process parameters used for the manufacturing of tensile and square specimens are listed in Table S1.

Table S1. Process parameters for LB-PBF-P. EOSINT P385 process parameters for manufacturing of PA12 and composite specimens.

Sample	Laser power (nominal) in W	Laser power (output) in W	Scanning speed in mm/s	Hatch distance in mm	Powder bed temperature in °C	Energy density in J/mm³
PA12	33	27	4500	0.4	171	0.150
PA12 + 0.005 vol% CNP (Coll)	33	27	4500	0.4	171	0.150
PA12 + 0.005 vol% CNP (DC)	33	27	4500	0.4	171	0.150
PA12 + 0.05 vol% CNP (Coll)	25	21	4500	0.4	169	0.115
PA12 + 0.05 vol% CNP (DC)	28	23	4500	0.4	169	0.127
PA12 + 0.05 vol% Ag (Coll)	33	27	4500	0.4	172	0.150

The powder properties of PA12 and composites, which are relevant for the powder recoating process in LB-PBF-P, are summarized in Table S2.

Table S2. Powder properties. Properties of PA12 powders and composites.

Sample	Particle size distribution (x_{area}; area of particle projection) in µm			Hausner ratio	Flow function ffc
	x10,3	x50,3	x90,3		
PA12 (washed)	44.2 ± 0.9	59.4 ± 1.0	77.3 ± 0.5	1.14 ± 0.02	12.3 ± 1.0
PA12 + 0.005 vol% CNP Coll	43.6 ± 0.6	58.6 ± 0.4	76.5 ± 0.4	1.12 ± 0.01	15.2 ± 1.5
PA12 + 0.05 vol% CNP Coll	44.3 ± 0.4	59.2 ± 0.9	75.9 ± 0.9	1.11 ± 0.02	12.4 ± 1.8
PA12 + 0.05 vol% Ag Coll	43.4 ± 1.0	58.6 ± 0.8	75.0 ± 0.5	1.13 ± 0.01	14.3 ± 1.1
PA12 (virgin)	43.7 ± 0.8	58.4 ± 0.9	74.9 ± 1.2	1.10 ± 0.03	17.2 ± 0.7
PA12 + 0.005 vol% CNP DC	44.4 ± 1.4	58.8 ± 0.5	77.2 ± 2.1	1.14 ± 0.01	9.0 ± 0.9
PA12 + 0.05 vol% CNP DC	41.5 ± 0.4	58.1 ± 0.6	75.8 ± 0.5	1.10 ± 0.02	8.3 ± 0.6

A summary of the lattice spacings of additively manufactured specimens measured by XRD can be found in Table S3.

Table S3. d-spacings (from XRD) of the manufactured specimens. Observed lattice spacings for the 100, 002 and 020 orientations for γ PA12 in composite specimens.

Material composition	d_{100} in nm	d_{002} in nm	d_{020} in nm
PA12	0.415	0.407	1.591
PA12 + 0.005 vol% CNP Coll	0.414	0.406	1.594
PA12 + 0.05 vol% CNP Coll	0.408	0.415	1.580
PA12 + 0.005 vol% CNP DC	0.414	0.406	1.598
PA12 + 0.05 vol% CNP DC	0.414	0.407	1.584
